# Associations among homocysteine, left cortical thickness, and working memory in patients with treatment-resistant schizophrenia: preliminary findings of a cohort study

**DOI:** 10.1186/s12888-026-08291-8

**Published:** 2026-06-13

**Authors:** Mengying Ma, Yanli Li, Jinghui Tong, Yanfang Zhou, Shuping Tan, Zhiren Wang, Fude Yang, Baopeng Tian, Junchao Huang, Yunlong Tan

**Affiliations:** Beijing Huilongguan Hospital, Capital Medical University, Peking University HuiLongGuan Clinical Medical School, Beijing, P. R. China

**Keywords:** Treatment-resistant schizophrenia, Homocysteine, Working memory, Cortical thickness, Suppression effect

## Abstract

**Background:**

Treatment-resistant schizophrenia (TRS) is characterized by severe cognitive impairments. However, the underlying mechanisms driving these deficits and strategies to mitigate them remain unclear. Elevated homocysteine levels have also been implicated in disease progression. We aimed to explore the relationship among serum homocysteine levels, working memory, and cortical thickness in patients with TRS.

**Methods:**

Eighty-five patients with schizophrenia (42 with TRS and 43 with non-TRS [NTRS]) and 58 healthy controls (HCs) were recruited. Serum homocysteine levels were measured using enzymatic cycling. Cortical thickness was assessed using FreeSurfer version 5.3. Mediation analysis was further performed to preliminarily explore the potential interplay among serum homocysteine levels, regional cortical thickness, and working memory performance. Given that the bivariate correlations between homocysteine levels and cortical thickness did not retain significance after false discovery rate (FDR) correction and the cross-sectional design, this analysis aimed to identify potential mediating or suppressive pathways for hypothesis-generating purposes, rather than verifying definite compensatory mechanisms.

**Results:**

Working memory was significantly impaired in patients with TRS and NTRS compared with HCs. Homocysteine levels were elevated in patients with schizophrenia, particularly in the TRS group. Among individuals with TRS, an inverse relationship was observed between serum homocysteine concentration and working memory (*r* = − 0.411, *p*_*FDR*_ = 0.02). Our exploratory suppression analyses revealed preliminary statistical links between elevated cortical thickness in the left precentral, postcentral, and precuneus gyri and homocysteine-associated working memory deficits in patients with TRS. These hypothesis-generating observations are constrained by non-significant FDR-corrected correlations and cross-sectional design.

**Conclusions:**

Our exploratory findings preliminarily indicate that greater cortical thickness within left-hemispheric brain regions may show statistical links to homocysteine-associated working memory deficits in patients with TRS, rather than definitive evidence of partial compensatory mechanisms. Notably, given the cross-sectional study design and non-significant bivariate correlations observed between homocysteine levels and cortical thickness following FDR correction, these results are strictly hypothesis-generating and cannot support causal inferences. The mediation-suppression analysis, performed despite non-significant FDR-adjusted pairwise associations, offers tentative clues regarding potential neural correlation patterns. Further longitudinal or interventional studies are required to validate these preliminary observations.

## Background

### Overview of schizophrenia, treatment-resistant schizophrenia, and homocysteine

Schizophrenia is a complex and debilitating psychiatric disorder characterized by disturbances in perception, cognition, and social functioning [1]. While traditional antipsychotic medications have demonstrated efficacy in managing symptoms for many individuals, a subset of patients exhibit resistance to standard treatments, leading to the distinct clinical entity known as treatment-resistant schizophrenia (TRS) [2]. The origin of schizophrenia involves intricate interactions among genetic, environmental, and neurobiological factors, with emerging research exploring the role of homocysteine, a sulfur-containing amino acid, in the pathophysiology of this condition [3].

### Working memory impairments in schizophrenia

Schizophrenia is a multifaceted disorder known for widespread neurocognitive and neuroanatomical impairments. Unfortunately, working memory impairments often show limited responsiveness to pharmacological interventions, which may contribute to the refractory nature observed in a subset of patients with schizophrenia (i.e., TRS). Notably, clozapine, the first-line treatment for TRS, is associated with weight gain, a common adverse reaction that may intersect with metabolic health and indirectly impact disease trajectories [4]. Furthermore, these working memory deficits are already present in individuals experiencing their first episode of psychosis [4, 5], suggesting that such impairments arise early in the illness course and cannot be fully explained by exposure to psychotropic medications. Antipsychotic drugs are theoretically expected to improve cognitive function in patients with schizophrenia; however, their clinical effects are often modest and inconsistent. This suggests that additional factors, including metabolic comorbidities, may also contribute to cognitive impairment in patients with TRS.

### Correlation between cortical thickness and working memory in schizophrenia

Some evidence indicates specific and nonspecific correlations between cortical thickness and working memory function in individuals with schizophrenia [6]. A clustering method was used to classify individuals with schizophrenia who exhibited significant cortical thinning, particularly in the fronto-temporal regions. This subgroup demonstrated more pronounced impairments in working memory [7]. Additionally, working memory performance in patients with schizophrenia may be related to the cortical thickness of the right superior temporal gyrus and right middle temporal gyrus. In contrast, working memory is associated with thickness of the lateral prefrontal cortex in the control group [8]. Previous research suggests that metabolic factors may also modulate the relationship between cortical thickness and cognitive function in schizophrenia [9], highlighting the complexity of neurocognitive impairment in this population.

### Homocysteine association with brain structure and executive function

Homocysteine is an intermediate product of methionine and cysteine metabolism. Early evidence indicates that elevated serum homocysteine levels are associated with asymptomatic cerebral infarctions, increased severity of periventricular white matter lesions, and expansion of subcortical white matter lesions [10]. Furthermore, the interaction between serum homocysteine levels and fractional anisotropy in the right cingulum and right inferior longitudinal fasciculus affects executive function in older individuals with depression [11]. Core executive functions include working memory [12]. Hence, serum homocysteine may influence individual brain structures and affect working memory function.

### Association between homocysteine and schizophrenia

Higher homocysteine levels have been observed in young male patients with schizophrenia [13], as well as in first-episode, unmedicated, and medicated patients with schizophrenia [14]. These findings suggest that changes in homocysteine levels may be one of the pathophysiological mechanisms underlying schizophrenia. Sex is recognized as an important determinant of circulating homocysteine levels, and emerging evidence supports a close link between hyperhomocysteinemia and metabolic syndrome, a highly prevalent comorbidity in individuals with schizophrenia [9]. While sex-related differences in homocysteine concentrations have been documented in schizophrenia cohorts [9, 13] and clozapine-associated weight gain may indirectly modulate metabolic profiles and homocysteine metabolism [15], the present study focused on the core relationships among homocysteine levels, cortical thickness, and working memory in patients with TRS. These interconnected metabolic and sex-related factors represent important contextual variables that warrant further investigation in future studies to clarify their potential modulatory roles in the observed neurocognitive and structural associations.

### Research gaps and study objectives

Elevated serum homocysteine levels are associated with compromised working memory [16]. The effects of high serum homocysteine levels on working memory function in patients with schizophrenia, particularly those with TRS, have not yet been fully elucidated. Additionally, the role of cortical thickness alterations in modulating impaired working memory in TRS remains unclarified. Given these gaps, we conducted this study to explore correlations among homocysteine levels, working memory, and cortical thickness in patients with TRS. While focusing on the potential link between homocysteine levels and working memory in patients with TRS, it is important to note that this study constitutes an exploratory investigation into whether cortical thickness may be involved in the statistical association between homocysteine levels and working memory in TRS. Notably, given the cross-sectional design, no causal inferences are intended. Furthermore, as bivariate correlations between homocysteine levels and cortical thickness did not survive false discovery rate (FDR) correction, the exploration of their potential correlational patterns was pursued to generate preliminary insights, with the understanding that such analyses should be interpreted as hypothesis-generating rather than confirmatory.

## Methods

### Participants

Overall, 90 inpatients diagnosed with schizophrenia per the Diagnostic and Statistical Manual of Mental Disorders, Fourth Edition criteria, were recruited from Beijing Huilongguan Hospital between 2017 and 2019. The participants were categorized into two groups: 45 patients with TRS and 45 with non-TRS (NTRS). Sixty HCs were enrolled via local advertisements. All participants voluntarily participated in the study. The exclusion criteria were a lifetime history of other psychiatric disorders, current or past substance dependence (excluding nicotine), head trauma, or serious systemic disorders. The Ethics Committee of Beijing Huilongguan Hospital approved the study protocol. All participants provided written informed consent.

TRS and NTRS were determined per established consensus guidelines [17]. Patients with TRS exhibited a minimal response to *≥* 6 weeks of treatment with *≥* 2 antipsychotic drugs, combined with ≥ 600 mg/day of chlorpromazine-equivalent, a total score of ≥ 45 on the Brief Psychiatric Rating Scale, and a score of ≥ 4 on the Clinical Global Impression-Severity of Illness (CGI-SI) scale. Patients with NTRS demonstrated periods of positive clinical response to antipsychotic medications and a CGI-SI score of < 3 for > 12 weeks.

### Clinical and cognitive assessment

A minimum of two experienced psychiatrists initially assessed all participants using the Brief Psychiatric Rating Scale and CGI-SI for group classification, followed by the Positive and Negative Syndrome Scale (PANSS) to evaluate schizophrenia symptoms. The inter-rater intra-class correlation coefficient exceeded 0.80. Cognitive assessment was performed using the MATRICS Consensus Cognitive Battery (MCCB), which encompasses seven cognitive domains and a composite score [17–19]. Transformation into Chinese normative T-scores was applied to convert the initial eight raw scores [19]. Our previous study demonstrated that working memory constitutes the primary cognitive domain that distinguishes individuals with TRS and NTRS [20].

### Serum sample collection and processing

Forty-four patients with TRS, 45 with NTRS, and all HCs underwent serum homocysteine level analysis. Approximately 3 mL of fasting blood was collected from each participant between 06:00 and 09:00 and stored in EDTA anticoagulant tubes. Inspection confirmed that all serum samples were hemolysis-free and had a chylous appearance. The samples were maintained at 2–8 °C and processed within 30 min. Using an AU5800 biochemical analyzer (Beckman Coulter Life Sciences, United States), serum homocysteine levels were determined using an enzymatic cycling method (Beijing Leadman Biotechnology, China). The detection parameters were as follows: first, 13 µL of the serum sample was pipetted into AU5800 biochemical analyzer. Reagent 1 was added, and the sample was maintained at 37 °C for 5 min. Reagent 2 was then added. After an additional 2.5 min at 37 °C, the absorbance value A1 was recorded, followed by A2 after an additional 5 min. The main wavelength was 340 nm, and the secondary wavelength was 700 nm. The reaction was based on the endpoint method, and the reaction direction was decreasing (−). The absorbance difference of the calibrator (A2-A1) was calculated, and a calibration curve for the absorbance versus concentration was constructed. The absorbance difference of the sample was determined, and the corresponding concentration from the calibration curve was obtained, which recorded the value. A homocysteine concentration of ≤ 15 µmol/L was considered within the normal reference range [21].

### Image processing

All participants underwent structural image scanning using a 3.0-T Prisma magnetic resonance imaging (MRI) scanner (MAGNETOM Prisma, Siemens, Munich, Germany) equipped with a 64-channel radio-frequency head coil at the Center for MRI Research, Beijing Huilongguan Hospital. Structural T1-weighted images were acquired using a three-dimensional-magnetization-prepared rapid gradient echo imaging sequence with the following parameters: repetition time/echo time, 2,530/2.98 ms; inversion time, 1,100 ms; flip angle, 7°; field of view, 256 × 224 mm^2^; thickness/gap, 1/0 mm; and matrix size, 256 × 224. Thirty-four regions of interest were delineated in each cerebral hemisphere, focusing on the cortical gray matter. The Desikan–Killiany atlas was used for the anatomical delineation of these regions [22, 23]. Subsequently, cortical thickness measurements were computed for each identified region, resulting in 68 cortical thickness values across both hemispheres. We used FreeSurfer version 5.3 (http://surfer.nmr.mgh.harvard.edu/) [24] for automated segmentation and validation of brain regions.

### Statistical analyses

SPSS 26.0 standard statistical package (IBM, Chicago) was used to analyze demographic and clinical data, including analysis of variance, χ^2^ test, and t-test. OriginPro 2021 (https://www.originlab.com/) and R version 4.3.1 (https://www.r-project.org/) were used to generate statistical figures. The relationships between homocysteine levels and brain cortical thickness (of 68 cortical regions) or cognitive function were determined using Pearson correlations. The relationship between events A and B may be mediated or suppressed by event C (mediator), which is considered the pathway or mechanism via which event A influences event B [25]. To test the hypothesis that brain structure (cortical thickness) mediates or suppresses the effect of serum homocysteine concentration on working memory, we employed the mediation package in R software for mediation analysis. This toolkit assesses indirect effects between dependent and independent variables (working memory) via the mediator (cortical thickness). Using a bootstrapping resampling strategy, we conducted 5,000 iterations to examine the mediation hypothesis. Statistical significance was set at *p* < 0.05 following FDR correction to minimize Type I errors. Specifically, FDR correction (Benjamini–Hochberg method) was applied across all 68 cortical regions as defined according to the Desikan–Killiany atlas to control for multiple comparisons in correlation analyses among homocysteine levels, cortical thickness, and working memory.

## Results

### Comparison of demographic and clinical data among the three groups

**Fig. 1:**
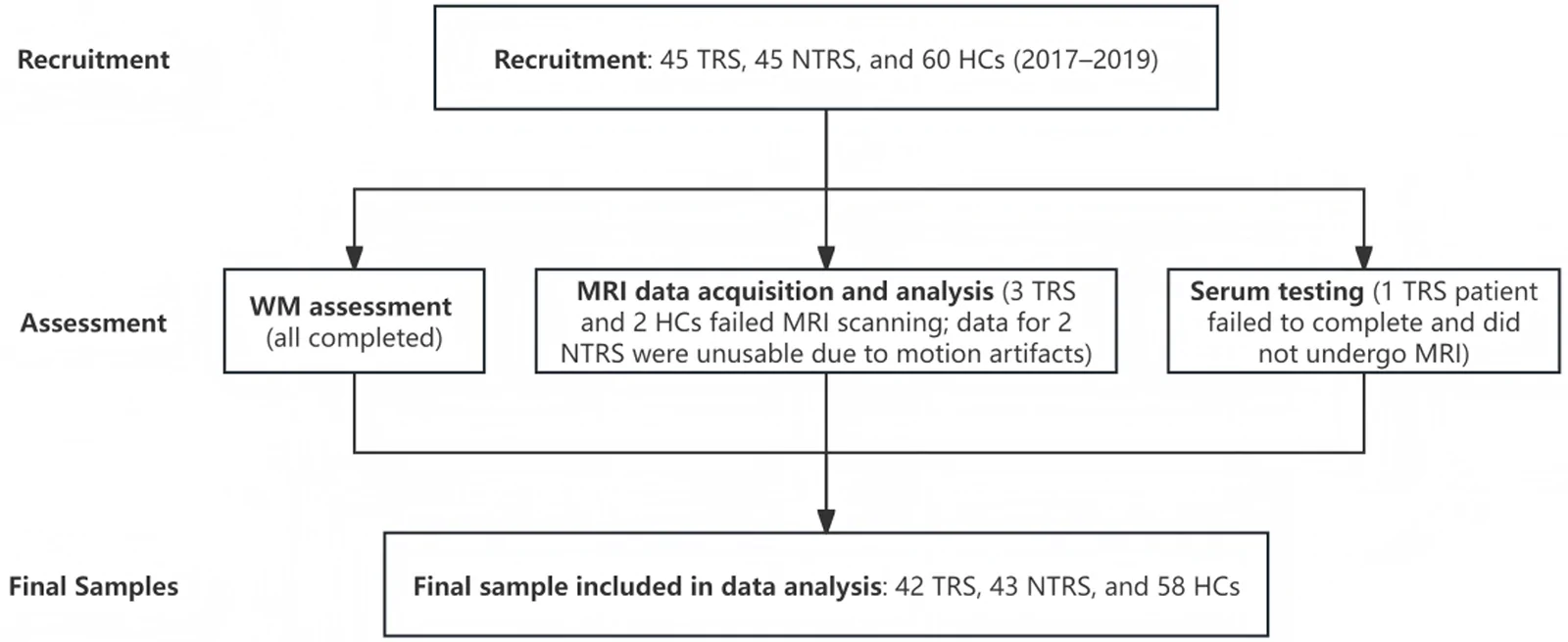
Flowchart of participants in the treatment-resistant schizophrenia study. TRS, treatment-resistant schizophrenia; NTRS, non-treatment-resistant schizophrenia; HCs, healthy controls; MRI, magnetic resonance imaging; WM, working memory

Three patients with TRS and two HCs were excluded from the analysis due to unsuccessful cooperation during MRI data acquisition. Two patients with NTRS were excluded from the data analysis because of significant motion artifacts in images. Serum samples were not successfully collected from one patient with TRS who did not undergo MRI. All participants completed the working memory assessment. Finally, 42 patients with TRS, 43 with NTRS, and 58 HCs were included in analyses. The process of participant recruitment and screening is presented in Fig. 1. The three groups were comparable in terms of age and sex distribution and educational levels (Table 1).

**Table 1:** Demographic and behavioral characteristics of the study population

	TRS (*n* = 42)	NTRS (*n* = 43)	HCs (*n* = 58)	F/χ^2^/t	*p*	*p*_FDR_
Age (yrs)	48.31 ± 8.46	46.26 ± 10.83	46.26 ± 8.03	0.76	0.47	0.47
Sex (female/male)	14/28	14/29	28/30	3.41	0.18	0.18
Education (yrs)	11.60 ± 2.72	11.28 ± 3.40	12.21 ± 2.51	1.38	0.26	0.38
Homocysteine level (µmol/L)	29.03 ± 16.51	25.59 ± 14.91	17.38 ± 12.86	8.09	< 0.001	< 0.001
Working memory score	39.52 ± 13.67	46.33 ± 10.68	55.19 ± 9.54	24.34	< 0.001	< 0.001
PANSS total score	85.02 ± 11.43	49.12 ± 10.94	-	14.8	< 0.001	< 0.001

Continuous variables are presented as means ± standard deviations. Group differences were tested using independent samples t-tests (for two groups) or one-way analysis of variance (for three groups), with *F/t* values reported. Categorical variables are presented as counts (percentages), and group differences were tested using chi-square (χ²) tests. TRS, treatment-resistant schizophrenia; NTRS, non-treatment-resistant schizophrenia; HCs, healthy controls; MCCB, MATRICS Consensus Cognitive Battery; PANSS, Positive and Negative Syndrome Scale; FDR, false discovery rate; Yrs, years.

**Fig. 2:**
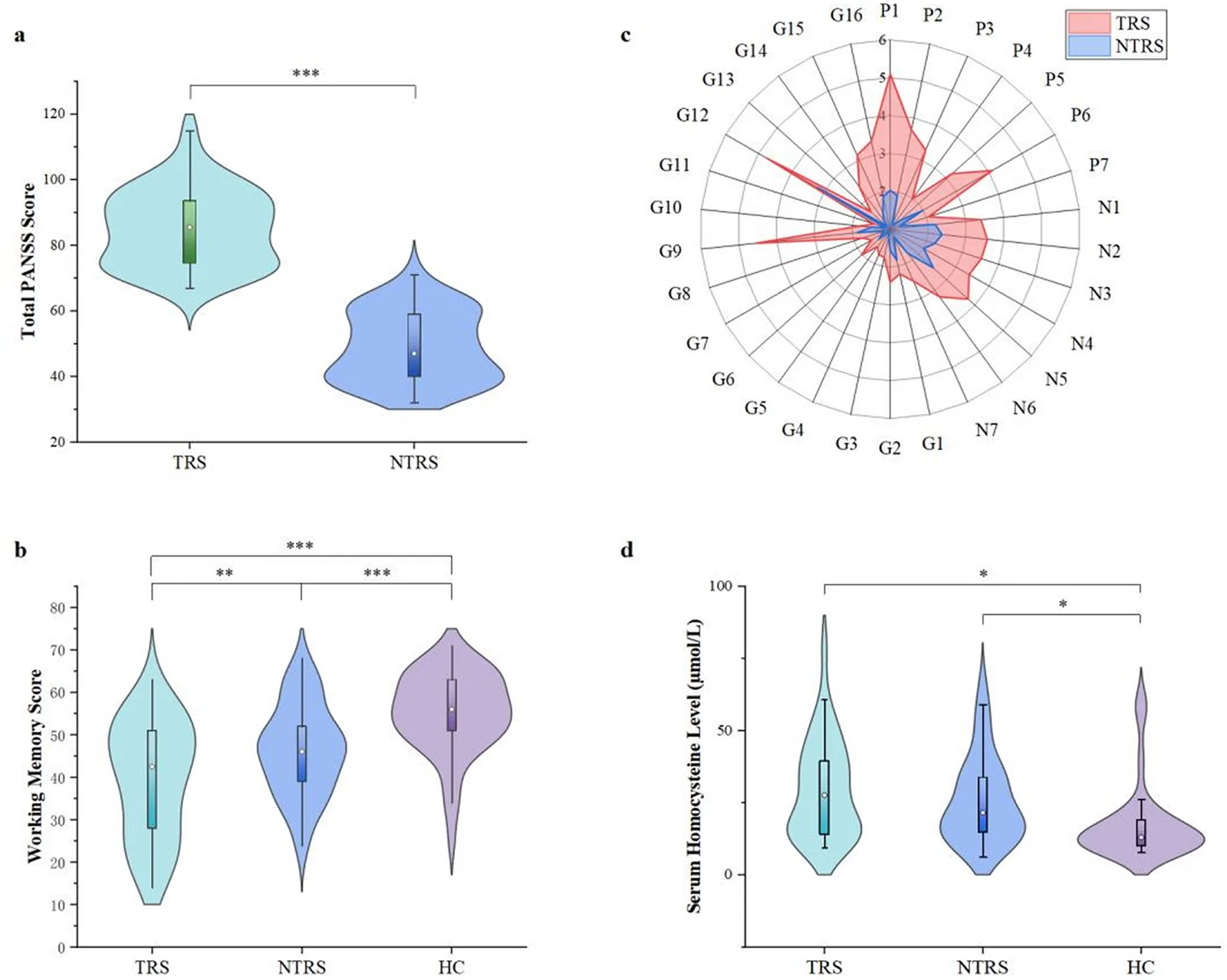
Comparison of PANSS score, working memory score, and serum homocysteine levels among the three groups. Panels **a** and **c** depict a notable discrepancy in symptom severity between the treatment-resistant schizophrenia (TRS) and non-treatment-resistant schizophrenia (NTRS) groups, as evidenced by the Positive and Negative Syndrome Scale (PANSS) scores. Specifically, individuals with TRS exhibited significantly higher symptom severity than individuals with NTRS. Panel **b** illustrates the pervasive cognitive impairment observed in both the NTRS and TRS groups compared with the healthy control (HC) group. Panel d indicates discernible inter-group variations in homocysteine levels among the TRS, NTRS, and HC groups

Given the lack of significant differences in age, sex, and education level among the three groups, MCCB scores were already adjusted for these variables. Subsequent analyses did not include age, sex, or education level as covariates. The mean PANSS score was significantly higher in the TRS group (85.02 ± 11.43) than in the NTRS group (49.12 ± 10.94; t = 14.8, *p*_*FDR*_ < 0.001), indicating that patients with TRS experienced more severe symptoms (Fig. 2a and c).

We observed that working memory was poorer in the NTRS (46.33 ± 10.68) and TRS (39.52 ± 13.67) groups than in the HC group (55.19 ± 9.54; F = 24.34, *p*_*FDR*_ < 0.001; Fig. 2b). Furthermore, greater deficits in working memory were observed in the TRS group than in the NTRS group (t = − 2.55, *p*_*FDR*_ = 0.01). Multiple comparison analyses indicated that homocysteine levels were significantly higher in the TRS and NTRS groups than in the HC group (F = 8.09, *p*_*FDR*_ < 0.001). However, the difference between the TRS and NTRS groups was non-significant (t = 1.01, *p*_*FDR*_ = 0.35; Fig. 2d).

Further comparison of cortical thickness between the groups revealed no significant differences in cortical thickness between the TRS and NTRS groups. However, the cortical thickness in the bilateral superior frontal gyrus (right hemisphere: TRS: 2.75 ± 0.1, HC: 2.92 ± 0.13, t = − 7.14, *p*_*FDR*_ < 0.001; left hemisphere: TRS: 2.58 ± 0.11, HC: 2.93 ± 0.13, t = − 6.61, *p*_*FDR*_ < 0.001), middle temporal gyrus (right hemisphere: TRS: 2.9 ± 0.16, HC: 3.08 ± 0.15, t = -6.03, *p*_*FDR*_ < 0.001; left hemisphere: TRS: 2.91 ± 0.15, HC 3.08 ± 0.16, t = -5.46, *p*_*FDR*_ < 0.001), and anterior cingulate gyrus and sulcus (right hemisphere: TRS: 2.52 ± 0.1, HC: 2.67 ± 0.14, t = -5.64, *p*_*FDR*_ < 0.001; left hemisphere: TRS: 2.59 ± 0.12, HC 2.76 ± 0.16, t = -6.14, *p*_*FDR*_ < 0.001) was significantly reduced in the TRS group compared with that in the HC group. Compared with those in the HC group, patients in the NTRS group exhibited reduced cortical thickness in the bilateral middle anterior cingulate gyrus and sulcus (right hemisphere: NTRS: 2.6 ± 0.15, HC: 2. 7 ± 0.11, t = -3.75, *p*_*FDR*_ < 0.001; left hemisphere: NTRS: 2.55 ± 0.15, HC 2.67 ± 0.14, t = -4.2, *p*_*FDR*_ < 0.001), bilateral superior frontal gyrus (right hemisphere: NTRS: 2.75 ± 0.1, HC: 2.92 ± 0.13, t = -4.96, *p*_*FDR*_ < 0.001; left hemisphere: NTRS: 2.81 ± 0.12, HC 2.93 ± 0.13, t = -4.63, *p*_*FDR*_ < 0.001), and right middle temporal gyrus (NTRS: 2.94 ± 0.15, HC: 3.08 ± 0.15, t = -4.88, *p*_*FDR*_ < 0.001).

### Correlations between homocysteine levels and working memory

**Fig. 3:**
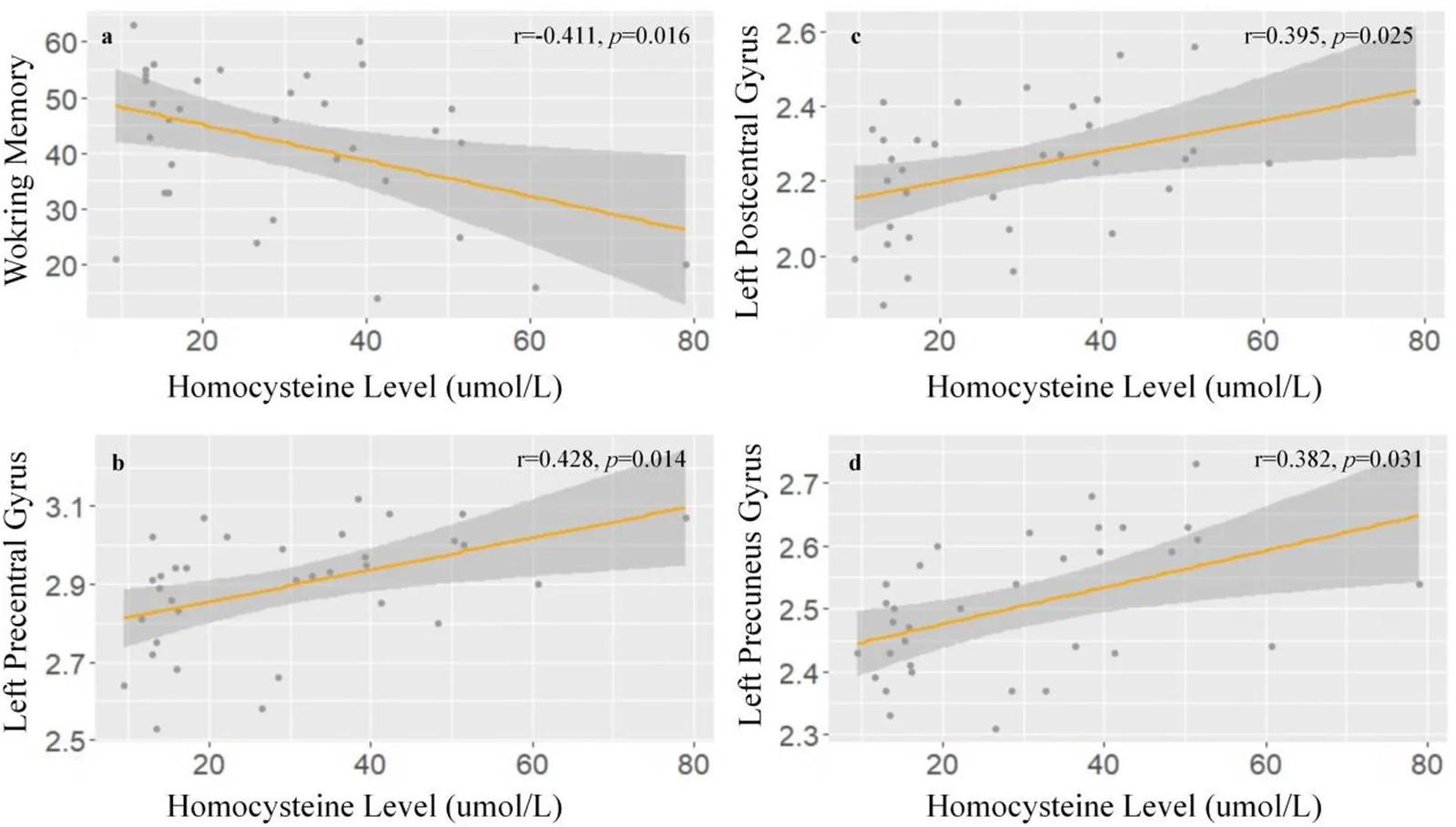
Association of serum homocysteine levels with working memory and cortical thickness in specific brain regions

Serum homocysteine levels were negatively correlated with working memory function (*r* = − 0.411, *p*_*FDR*_ = 0.02) in the TRS group (Fig. 3a). However, no significant correlations were observed between serum homocysteine levels and working memory in either the NTRS or HC group.

### Correlations between homocysteine levels and cortical thickness

Controlling for the confounding effects of age and sex and further accounting for multiple comparisons via FDR correction, no significant correlation was observed between homocysteine levels and cortical thickness in this cohort. However, some results showed trends suggesting a potential association. Specifically, positive correlations were observed between serum homocysteine levels and cortical thickness of the precentral gyrus (*r* = 0.428, *p*_unadjusted_ = 0.01) (Fig. 3b), postcentral gyrus (*r* = 0.395, *p*_unadjusted_ = 0.03) (Fig. 3c), and precuneus gyrus (*r* = 0.382, *p*_unadjusted_ = 0.03) (Fig. 3d) in the left hemispheres of patients with TRS.

Positive correlations were also observed between serum homocysteine levels and cortical thickness of the left superior parietal gyrus (*r* = 0.42, *p*_unadjusted_ < 0.01), left temporalis superior lateral (*r* = 0.401, *p*_unadjusted_ = 0.01), left frontal superior sulcus (*r* = 0.334, *p*_unadjusted_ = 0.04), right precuneus gyrus (*r* = 0.349, *p*_unadjusted_ = 0.03), and right subparietal sulcus (*r* = 0.366, *p*_unadjusted_ = 0.02) in patients with NTRS.

Negative correlations were observed between serum homocysteine levels and cortical thickness of the bilateral lingual portions of the left central sulcus (*r* = -0.416, *p*_unadjusted_ = 0.02), right dorsal posterior cingulate gyrus (*r* = -0.347, *p*_unadjusted_ = 0.05), and right pericallosal sulcus (*r* = -0.355, *p*_unadjusted_ = 0.04) in the HC group.

### Correlations between working memory and cortical thickness

No significant correlation between working memory and cortical thickness was observed after applying FDR correction. Nevertheless, several results indicated trends that may suggest a possible connection. Specifically, a positive correlation was observed between working memory and cortical thickness of the left paracentral gyrus (*r* = 0.36, *p*_*unadjusted*_ = 0.04) in the TRS group. Working memory was also positively correlated with cortical thickness of the following brain areas in the NTRS group: the left frontomarginal gyrus (*r* = 0.4, *p*_*unadjusted*_ = 0.01), left post-dorsal cingulate gyrus (*r* = 0.43, *p*_*unadjusted*_ < 0.01), left superior frontal gyrus (*r* = 0.38, *p*_*unadjusted*_ = 0.02), left precentral gyrus (*r* = 0.44, *p*_*unadjusted*_ < 0.01), left precuneus gyrus (*r* = 0.32, *p*_*unadjusted*_ = 0.05), central sulcus (*r* = 0.43, *p*_*unadjusted*_ < 0.01), left inferior frontal sulcus (*r* = 0.37, *p*_*unadjusted*_ = 0.02), and left middle frontal sulcus (*r* = 0.38, *p*_*unadjusted*_ = 0.02). In the HC group, negative correlations were observed between working memory and cortical thickness in the left post-dorsal cingulate gyrus (*r* = − 0.33, *p*_*unadjusted*_ = 0.01) and bilateral middle frontal gyrus (left *r* = − 0.43, *p*_*unadjusted*_ < 0.01; right *r* = − 0.38, *p*_*unadjusted*_ < 0.01).

### Relationships among homocysteine levels, working memory, and cortical thickness

**Fig. 4:**
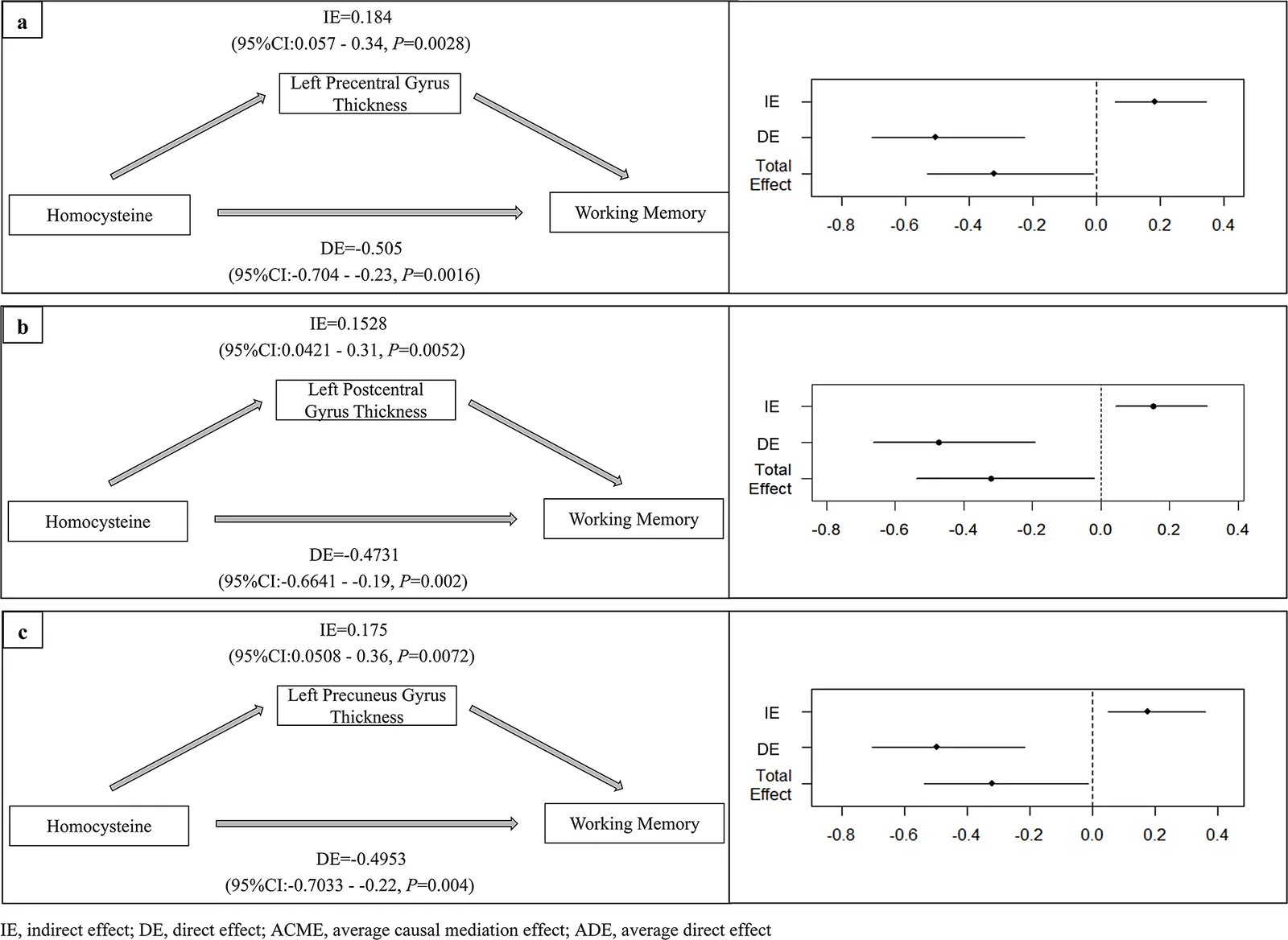
Flow diagram of the relationship among homocysteine levels, cortical thickness, and working memory

The present causal mediation analysis, performed with 5,000 nonparametric bootstrap simulations, preliminarily explored potential underlying associations among serum homocysteine levels, regional cortical thickness, and working memory performance in patients with TRS. Consistent with the statistical characteristics of mediation models, obvious suppression effects were observed in three core brain regions. Specifically, the average causal mediation effect was significant in the left precentral gyrus (estimate = 0.184, 95% confidence interval [CI]: 0.057–0.34, *p* = 0.0028), left postcentral gyrus (estimate = 0.1528, 95% CI: 0.0421–0.31, *p* = 0.0052), and left precuneus (estimate = 0.1750, 95% CI: 0.0508–0.36, *p* = 0.0072) (Fig. 4). Correspondingly, significant negative average direct effects and significant total effects were also identified across all three cortical regions. Notably, the calculated proportion of mediated effects presented negative values with extremely wide bootstrap confidence intervals in all analyses, further indicating a typical suppression pathway, rather than a conventional mediating relationship. Considering the limited sample size of the current study and cross-sectional design, such significant statistical associations should be interpreted strictly as exploratory preliminary findings. We merely speculate that elevated peripheral homocysteine levels may correlate with poor working memory function in TRS, and increased cortical thickness in the abovementioned left brain regions may exhibit a potential preliminary compensatory tendency in such biological correlations. No definite causal inference or deterministic regulatory mechanism can be concluded from the current statistical results. In addition to the indirect suppressive pathway, homocysteine was also found to exert a direct statistical influence on working memory performance in the enrolled cohort of patients with TRS (Fig. 4). However, no such effect was observed in either the NTRS or HC group.

This graphical representation illustrates the hypothetical correlational patterns via which homocysteine levels may be linked to working memory in the treatment-resistant schizophrenia group: increased cortical thickness of the left precentral, postcentral, and precuneus gyri may be involved in the statistical association between elevated homocysteine levels and working memory performance.

## Discussion

To the best of our knowledge, this is the first study to identify increased cortical thickness in the left precentral gyrus, postcentral gyrus, and precuneus in patients with TRS, which may reflect a tentative correlational pattern related to working memory impairments associated with elevated serum homocysteine levels. In patients with TRS, higher serum homocysteine levels were associated with a more pronounced decline in working memory function. Concurrently, a corresponding increase in cortical thickness was observed in these three brain regions, suggesting a potential statistical association between homocysteine levels, structural brain changes, and cognitive performance in this patient population. Our exploratory findings provide preliminary clues regarding how left-hemispheric cortical thickness may correlate with homocysteine-related working memory deficits in patients with TRS. As our preliminary cohort study adopted a cross-sectional design, we aimed to generate hypotheses regarding the correlational pathways linking homocysteine levels, cortical thickness, and working memory, despite the failure of bivariate correlations to retain statistical significance after strict FDR multiple comparison correction. We, therefore, interpret the mediation-suppression results with great caution, as these observations are hypothesis-generating, rather than confirmatory evidence, requiring further validation in larger-sample and longitudinal studies.

### Potential mechanisms through which elevated homocysteine levels affect working memory in patients with TRS

Animal studies have demonstrated that elevated homocysteine levels can impair spatial memory in rats [26]. Homocysteine induces neuronal cell death in primary cultures of cerebellar granule cells from mice by activating *N*-methyl-d-aspartate (NMDA) receptors and generating free radicals [27]. Homocysteine may be produced during dopamine degradation and could enhance NMDA receptor function by reducing receptor desensitization. This enhanced NMDA receptor activity is crucial for sustained neural activity, such as working memory. However, excessively high homocysteine levels may prevent normal receptor desensitization, leading to overactivation of neural circuits. This could result in working memory errors or persistent memory interference, which is particularly evident in conditions such as schizophrenia. This suggests that homocysteine may play a regulatory role in brain function, particularly in working memory, and may be linked to the cognitive deficits in working memory observed in schizophrenia [16].

Elevated homocysteine levels may also alter neurotrophic factor levels in the brain, which are crucial for neuronal survival and function, further affecting working memory. Elevated levels of homocysteine impair both short- and long-term memory, and reduce the levels of endogenous neurotrophic factors in the hippocampi of Wistar rats. Pretreatment of Wistar rats with folic acid prevents memory deficits induced by homocysteine injection and the associated decrease in brain-derived neurotrophic factor levels [28].

Notably, longitudinal studies have highlighted distinct, long-term cognitive trajectories between TRS and non-TRS schizophrenia, with TRS (especially ultra-treatment resistant) associated with more severe and progressive cognitive decline than treatment-responsive subgroups [29]. Given homocysteine’s role in mediating neurotoxic pathways (e.g., NMDA receptor dysfunction, neurotrophic factor reduction) linked to cognitive deterioration, it is plausible that sustained elevated homocysteine levels contribute to persistent cognitive impairment and long-term decline in TRS. This supports the theory that treatment resistance and cognitive decline may share a common neurobiological substrate [29], with homocysteine potentially acting as a key mediator. Additionally, metabolic syndrome (MetS), a highly prevalent comorbidity in schizophrenia, and the kynurenine pathway may form a synergistic network with homocysteine to exacerbate cognitive impairment in TRS [30]. MetS-related insulin resistance and low-grade inflammation can disrupt homocysteine metabolism while also activating the kynurenine pathway to produce neurotoxic metabolites (e.g., quinolinic acid) that interact with homocysteine to amplify neural damage. These interconnected pathways may further reinforce the refractory nature of cognitive deficits in TRS, although detailed exploration of these interactions could be conducted in future studies.

### Relationship between the left precentral, postcentral, and precuneus gyri and working memory

The pre- and postcentral gyri are critical regions of the primary motor and somatosensory cortices. Their primary function is to receive, integrate, and respond to external sensory-motor stimuli. The inhibition of neural activity in these regions may delay the transmission of motor signals within motor pathways, disrupt the coordination between sensory input and motor output, and further lead to impairments in executive control functions and, consequently, weakening cognitive functions related to working memory [31], such as information manipulation and response execution. The left precentral gyrus may serve as the primary brain region that maintains verbal working memory in response to auditory stimuli [32]. The left precentral and postcentral gyri are important components of the attention network [33] and are essential for filtering and prioritizing sensory information, directing attention to relevant stimuli. The functional state of working memory appears to be closely intertwined with an individual’s ability to sustain attention. Additionally, the interaction between the pre- and postcentral gyri via U-shaped fibers may facilitate the modulation of working memory [32]. Studies on working memory in healthy adolescents have demonstrated that neural activation in the left precuneus increases with age [34]. This brain region is frequently mentioned in working memory research [35] and rule-guided conditional reasoning operations, which require extensive cognitive resources to maintain working memory [36, 37]. Additionally, the precuneus is linked to representation and processing of spatial relationships among different object features [38]. The precuneus significantly facilitates enhanced domain-general executive processes, such as the rapid switching of attention [39]. The ability to shift attention is a component of several executive processes that control working memory. This includes sustaining information in working memory while simultaneously processing additional data, manipulating that information within working memory, and determining which pieces of information from multiple sources should be retained. These tasks are complex and necessitate transitions between sensory input and information already held in working memory [34]. In summary, the left precentral, postcentral, and precuneus gyri may play crucial roles in modulating working memory through sensory-motor integration, attentional control, and executive function regulation. The structural and functional integrity of these regions is closely linked to working memory performance, and their impairment may contribute to cognitive deficits in conditions such as TRS. Our exploratory observations suggest that greater cortical thickness in these brain regions may be statistically associated with homocysteine-related working memory deficits, rather than acting as definitive compensatory adaptations. Such potential structural changes may reflect hypothetical neuroplastic patterns in response to pathological alterations, which could preliminarily maintain cognitive function, to a certain extent. Given the cross-sectional design and non-significant bivariate correlations after FDR correction, this interpretation remains hypothesis-generating and requires further verification.

### Preliminary exploration of left cortical thickness correlates with homocysteine-related working memory impairment in TRS

In patients with TRS, greater cortical thickness within the left precentral gyrus, postcentral gyrus, and left precuneus may represent tentative structural correlates linked to homocysteine-associated working memory decline, rather than definitive compensatory brain adaptation. Older adults with superior cognitive performance tend to exhibit thicker cortical thickness in specific brain regions, including the left anterior cingulate cortex [40]. This observation suggests that regional cortical thickening may correlate with preserved cognitive performance in certain populations. In patients with TRS, in whom cognitive impairments are pronounced, significant dysfunction in the prefrontal cortex may prompt the recruitment of other brain regions to meet cognitive demands [8]. Accordingly, the increased cortical thickness identified in the left precentral gyrus, postcentral gyrus, and precuneus observed here shows preliminary statistical associations with homocysteine-related working memory deficits. This regional pattern, observed specifically in patients with TRS, may imply hypothetical neural plasticity-related patterns, rather than proven compensatory processes. Such patients may engage a broader network of cortical regions to cope with prefrontal cortex dysfunction. Consequently, individuals with TRS may rely more heavily on cortical areas outside the lateral prefrontal cortex to maintain working memory function. However, over-engagement of these thickened regions may contribute to functional decline in other cognitive domains or increased neural fatigue. Additionally, increased cortical thickness cannot be solely interpreted as a compensatory response; it may also reflect pathological alterations, such as neuronal hyperexcitability or abnormal neural connectivity. Genes linked to cell migration, proliferation, axonal growth, myelination, synaptogenesis, and apoptosis, all of which are implicated in schizophrenia, may interact with environmental factors to induce neurodevelopmental abnormalities, including altered cortical layer organization [8]. These abnormalities may ultimately lead to the activation of atypical neural circuits [8]. Given our cross-sectional design and non-significant bivariate correlations between homocysteine levels and cortical thickness following FDR correction, all the abovementioned interpretations remain hypothesis-generating and require further longitudinal validation.

### Cortical thickness alterations in patients with TRS: comparisons with patients with NTRS and HCs

Numerous studies in the field of neuroimaging have explored brain structural alterations in patients with TRS. Most studies have demonstrated that, compared with HCs and patients with NTRS, patients with TRS exhibit cortical thinning or reduced gray matter volume in multiple core brain regions, including the frontal lobe, temporal lobe, parietal lobe, cerebellum, and insula [41–43]. Such structural abnormalities are widely regarded as being closely associated with disease severity, the development of treatment resistance, and cognitive impairment [41–43]. The present findings revealed that patients with TRS had significantly reduced cortical thickness in the bilateral superior frontal gyrus, middle temporal gyrus, and anterior cingulate gyrus and sulcus compared with HCs. Similar to patients with TRS, patients with NTRS also displayed reduced cortical thickness in the bilateral middle anterior cingulate gyrus and sulcus, bilateral superior frontal gyrus, and right middle temporal gyrus brain regions than HCs; however, no significant differences in cortical thickness were observed between the TRS and NTRS groups.

These results suggest that patients with TRS exhibit reduced brain volume in multiple regions, which may be associated with degenerative structural changes induced by long-term TRS disease progression. Notably, the present study focuses on the potential compensatory structural remodeling that may occur during the disease course of TRS, a direction that has not received sufficient attention in previous studies. Currently, research on the dynamic characteristics of such “coexistence of damage and compensation” in brain structure remains limited.

The current findings not only further corroborate previous conclusions regarding brain structural atrophy in patients with TRS but also suggest that brain structural changes in TRS are not a single degenerative process; rather, they represent a dynamic outcome of the interaction between degenerative damage and compensatory remodeling. This compensatory cortical thickening may represent an adaptive response by the brain to maintain basic cognitive function under long-term pathological stress, and its compensatory efficacy may be related to serum homocysteine levels, disease severity, and treatment response. Future studies should adopt a longitudinal follow-up design to further explore the compensatory mechanism and its association with the long-term prognosis of patients with TRS.

### Limitations

Some limitations warrant mention. First, the relatively small sample may have undermined the ability to detect significant differences in serum homocysteine levels between patients with TRS and NTRS, as well as in the cortical thickness of three specific brain regions: the left precentral gyrus, postcentral gyrus, and precuneus. Consistent with this, the non-significant correlations between homocysteine levels and cortical thickness following FDR correction for multiple comparisons may impact the reliability of the mediation analyses conducted in this study. Future studies with larger samples are, therefore, warranted to validate the results of the current study and further explore the potential mechanisms underlying the relationships among homocysteine levels, cortical thickness, and cognitive function in patients with schizophrenia. Second, this study employed a cross-sectional design, which inherently precludes the establishment of causal relationships, given that mediation analyses based on cross-sectional data cannot confirm directional or causal associations between variables. A longitudinal design, incorporating data collection at different stages of illness (e.g., onset, acute phase, and chronic phase), would provide better insight into the potential modulatory effect of serum homocysteine on brain structure and function, and help clarify the directional relationships among the variables of interest. Third, the relatively low coefficient of determination indicated that additional unmeasured confounding factors may also influence working memory performance.

## Conclusions

Our findings provide new insights into the potential biological underpinnings of working memory impairments in patients with TRS, as well as the associated hypothetical structural correlates involved, particularly those related to structural brain changes. Specifically, structural MRI analyses revealed that patients with TRS exhibit increased cortical thickness in the left hemisphere, which showed preliminary statistical associations with homocysteine-related working memory deficits, rather than serving as a definitive mechanism to offset such deficits. Importantly, this observed structural pattern was not linked to complete mitigation of the observed memory impairments, highlighting the limited role of this structural change in modifying cognitive deficits.

In summary, these findings contribute to our understanding of the potential biological processes associated with working memory deficits observed in patients with TRS while also offering valuable perspectives on the structural components that may correlate with such cognitive impairments. Our results tentatively suggest potential exploratory directions for future interventions focused on these structural correlates, which may ultimately help improve cognitive function and quality of life for individuals with TRS. These interpretations should be considered preliminary and hypothesis-generating, given the study’s methodological limitations, including its cross-sectional design and non-significant bivariate correlations between homocysteine levels and cortical thickness following FDR correction, and require further validation in larger, longitudinal cohorts.

## Data Availability

All pertinent data are contained within this manuscript.

## Abbreviations

TRS: Treatment-resistant schizophrenia
NTRS: Non-treatment-resistant schizophrenia
HC: Healthy control
CGI-SI: Clinical Global Impression-Severity of Illness
PANSS: Positive and Negative Syndrome Scale
FDR: False discovery rate
CI: Confidence interval
MCCB: MATRICS Consensus Cognitive Battery
MRI: Magnetic resonance imaging
NMDA: *N*-methyl-d-aspartate
MetS: Metabolic syndrome

## References

[1] Kronick J, Sabesan P, Burhan AM, Palaniyappan L. Assessment of treatment resistance criteria in non-invasive brain stimulation studies of schizophrenia. Schizophr Res. 2022;243:349–60. 10.1016/j.schres.2021.06.009. 34183208 10.1016/j.schres.2021.06.009

[2] Howes OD, mccutcheon R, Agid O, de Bartolomeis A, van Beveren NJM, Birnbaum ML, et al. Treatment-resistant schizophrenia: treatment response and resistance in psychosis (TRRIP) working group consensus guidelines on diagnosis and terminology. Am J Psychiatry. 2017;174:216–29. 10.1176/appi.ajp.2016.16050503. 27919182 10.1176/appi.ajp.2016.16050503 PMC6231547

[3] Muntjewerff JW, Kahn RS, Blom HJ, den Heijer M. Homocysteine, methylenetetrahydrofolate reductase and risk of schizophrenia: a meta-analysis. Mol Psychiatry. 2006;11:143–9. 10.1038/sj.mp.4001746. 16172608 10.1038/sj.mp.4001746

[4] Oomen PP, Begemann MJH, Brand BA, de Haan L, Veling W, Koops S, et al. Longitudinal clinical and functional outcome in distinct cognitive subgroups of first-episode psychosis: a cluster analysis. Psychol Med. 2023;53:2317–27. 10.1017/S0033291721004153. 34664546 10.1017/S0033291721004153 PMC10123843

[5] Liu X, Li Y, Xu L, Zhang T, Cui H, Wei Y, et al. Spatial and temporal abnormalities of spontaneous fixational saccades and their correlates with positive and cognitive symptoms in schizophrenia. Schizophr Bull. 2024;50:78–88. 10.1093/schbul/sbad039. 37066730 10.1093/schbul/sbad039 PMC10754167

[6] Alkan E, Davies G, Evans SL. Cognitive impairment in schizophrenia: relationships with cortical thickness in fronto-temporal regions, and dissociability from symptom severity. Npj Schizophr. 2021;7:1–9. 10.1038/s41537-021-00149-0. 33737508 10.1038/s41537-021-00149-0 PMC7973472

[7] Pan Y, Pu W, Chen X, Huang X, Cai Y, Tao H, et al. Morphological profiling of schizophrenia: cluster analysis of MRI-based cortical thickness data. Schizophr Bull. 2020;46:623–32. 10.1093/schbul/sbz112. 31901940 10.1093/schbul/sbz112 PMC7147597

[8] Ehrlich S, Brauns S, Yendiki A, Ho B-C, Calhoun V, Schulz SC, et al. Associations of cortical thickness and cognition in patients with schizophrenia and healthy controls. Schizophr Bull. 2012;38:1050–62. 10.1093/schbul/sbr018. 21436318 10.1093/schbul/sbr018 PMC3446215

[9] Bosia M, Spangaro M, Sapienza J, Martini F, Civardi S, Buonocore M, et al. Cognition in schizophrenia: modeling the interplay between interleukin-1β C-511T polymorphism, metabolic syndrome, and sex. Neuropsychobiology. 2021;80:321–32. 10.1159/000512082. 33395686 10.1159/000512082

[10] Vermeer SE, van Dijk EJ, Koudstaal PJ, Oudkerk M, Hofman A, Clarke R, et al. Homocysteine, silent brain infarcts, and white matter lesions: the Rotterdam Scan Study. Ann Neurol. 2002;51:285–9. 10.1002/ana.10111. 11891822 10.1002/ana.10111

[11] Zhou H, Zhong X, Chen B, Wang Q, Zhang M, Mai N, et al. Elevated homocysteine levels, white matter abnormalities and cognitive impairment in patients with late-life depression. Front Aging Neurosci. 2022;14:931560. 10.3389/fnagi.2022.931560. 35923546 10.3389/fnagi.2022.931560 PMC9340773

[12] Diamond A. Executive functions. Annu Rev Psychol. 2013;64:135–68. 10.1146/annurev-psych-113011-143750. 23020641 10.1146/annurev-psych-113011-143750 PMC4084861

[13] Pringle CR. Temperature-sensitive mutant vaccines. Methods Mol Med. 1996;4:17–32. 10.1385/0-89603-334-1:17. 21359692 10.1385/0-89603-334-1:17

[14] Berry T, Abohamza E, Moustafa AA. Treatment-resistant schizophrenia: focus on the transsulfuration pathway. Rev Neurosci. 2020;31:219–32. 10.1515/revneuro-2019-0057. 31714892 10.1515/revneuro-2019-0057

[15] Van Der Horst MZ, Meijer Y, De Boer N, Guloksuz S, Hasan A, Siskind D, et al. Comprehensive dissection of prevalence rates, sex differences, and blood level-dependencies of clozapine-associated adverse drug reactions. Psychiatry Res. 2023;330:115539. 10.1016/j.psychres.2023.115539. 37988817 10.1016/j.psychres.2023.115539

[16] Bolton AD, Constantine-Paton M. Synaptic effects of dopamine breakdown and their relation to schizophrenia-linked working memory deficits. Front Synaptic Neurosci. 2018;10:16. 10.3389/fnsyn.2018.00016. 29950984 10.3389/fnsyn.2018.00016 PMC6008544

[17] Kern RS, Nuechterlein KH, Green MF, Baade LE, Fenton WS, Gold JM, et al. The MATRICS Consensus Cognitive Battery, part 2: co-norming and standardization. Am J Psychiatry. 2008;165:214–20. 10.1176/appi.ajp.2007.07010043. 18172018 10.1176/appi.ajp.2007.07010043

[18] Nuechterlein KH, Green MF, Kern RS, Baade LE, Barch DM, Cohen JD, et al. The MATRICS Consensus Cognitive Battery, part 1: test selection, reliability, and validity. Am J Psychiatry. 2008;165:203–13. 10.1176/appi.ajp.2007.07010042. 18172019 10.1176/appi.ajp.2007.07010042

[19] Zou YZ, Cui JF, Wang J, Chen N, Tan SP, Zhang D, et al. Clinical reliability and validity of the Chinese version of measurement and treatment research to improve cognition in schizophrenia consensus cognitive battery. Chin J Psychiatry. 2009;42:29–33.

[20] Huang J, Zhu Y, Fan F, Chen S, Hong Y, Cui Y, et al. Hippocampus and cognitive domain deficits in treatment-resistant schizophrenia: a comparison with matched treatment-responsive patients and healthy controls★★★. Psychiatry Res Neuroimaging. 2020;297:111043. 10.1016/j.pscychresns.2020.111043. 32062167 10.1016/j.pscychresns.2020.111043 PMC7490244

[21] Ramires Junior OV, Dos Santos TM, Silveira JS, Leite-Aguiar R, Coutinho-Silva R, Savio LEB, et al. Rivastigmine reverses the decrease in synapsin and memory caused by homocysteine: is there relation to inflammation? Mol Neurobiol. 2022;59:4517–34. 10.1007/s12035-022-02871-x. 35578101 10.1007/s12035-022-02871-x

[22] Wong TY, Radua J, Pomarol-Clotet E, Salvador R, Albajes-Eizagirre A, Solanes A, et al. An overlapping pattern of cerebral cortical thinning is associated with both positive symptoms and aggression in schizophrenia via the ENIGMA consortium. Psychol Med. 2020;50:2034–45. 10.1017/S0033291719002149. 31615588 10.1017/S0033291719002149 PMC13196894

[23] Desikan RS, Ségonne F, Fischl B, Quinn BT, Dickerson BC, Blacker D, et al. An automated labeling system for subdividing the human cerebral cortex on MRI scans into gyral based regions of interest. NeuroImage. 2006;31:968–80. 10.1016/j.neuroimage.2006.01.021. 16530430 10.1016/j.neuroimage.2006.01.021

[24] Fischl B, Salat DH, Busa E, Albert M, Dieterich M, Haselgrove C, et al. Whole brain segmentation: automated labeling of neuroanatomical structures in the human brain. Neuron. 2002;33:341–55. 10.1016/S0896-6273(02)00569-X. 11832223 10.1016/s0896-6273(02)00569-x

[25] Baron RM, Kenny DA. The moderator-mediator variable distinction in social psychological research: conceptual, strategic, and statistical considerations. J Pers Soc Psychol. 1986;51:1173–82. 10.1037//0022-3514.51.6.1173. 3806354

[26] Matté C, Scherer EBS, Stefanello FM, Barschak AG, Vargas CR, Netto CA, et al. Concurrent folate treatment prevents Na+,K+-atpase activity inhibition and memory impairments caused by chronic hyperhomocysteinemia during rat development. Int J Dev Neurosci Off J Int Soc Dev Neurosci. 2007;25:545–52. 10.1016/j.ijdevneu.2007.10.003. 10.1016/j.ijdevneu.2007.10.003 18023318

[27] Kim WK, Pae YS. Involvement of N-methyl-d-aspartate receptor and free radical in homocysteine-mediated toxicity on rat cerebellar granule cells in culture. Neurosci Lett. 1996;216:117–20. 10.1016/0304-3940(96)13011-1. 8904797 10.1016/0304-3940(96)13011-1

[28] Matté C, Pereira LO, Dos Santos TM, Mackedanz V, Cunha AA, Netto CA, et al. Acute homocysteine administration impairs memory consolidation on inhibitory avoidance task and decreases hippocampal brain-derived neurotrophic factor immunocontent: prevention by folic acid treatment. Neuroscience. 2009;163:1039–45. 10.1016/j.neuroscience.2009.07.023. 19619620 10.1016/j.neuroscience.2009.07.023

[29] Spangaro M, Martini F, Bechi M, Buonocore M, Agostoni G, Cocchi F, et al. Longitudinal course of cognition in schizophrenia: does treatment resistance play a role? J Psychiatr Res. 2021;141:346–52. 10.1016/j.jpsychires.2021.07.019. 34304039 10.1016/j.jpsychires.2021.07.019

[30] Sapienza J, Agostoni G, Comai S, Nasini S, Dall’Acqua S, Sut S, et al. Neuroinflammation and kynurenines in schizophrenia: impact on cognition depending on cognitive functioning and modulatory properties in relation to cognitive remediation and aerobic exercise. Schizophr Res Cogn. 2024;38:100328. 10.1016/j.scog.2024.100328. 39281320 10.1016/j.scog.2024.100328 PMC11399803

[31] Chen X, Liu J, Wang J, Xin Z, Zhang Q, Zhang W, et al. Altered resting-state networks may explain the executive impairment in young health immigrants into high-altitude area. Brain Imaging Behav. 2021;15:147–56. 10.1007/s11682-019-00241-1. 32125618 10.1007/s11682-019-00241-1

[32] Kambara T, Brown EC, Jeong J-W, Ofen N, Nakai Y, Asano E. Spatio-temporal dynamics of working memory maintenance and scanning of verbal information. Clin Neurophysiol Off J Int Fed Clin Neurophysiol. 2017;128:882–91. 10.1016/j.clinph.2017.03.005. 10.1016/j.clinph.2017.03.005 PMC5429980 28399442

[33] Maimaitiaili S, Tang C, Liu C, Lv X, Chen Z, Zhang M, et al. Alterations in brain morphology and functional connectivity mediate cognitive decline in carotid atherosclerotic stenosis. Front Aging Neurosci. 2024;16:1395911. 10.3389/fnagi.2024.1395911. 38974904 10.3389/fnagi.2024.1395911 PMC11225314

[34] Andre J, Picchioni M, Zhang R, Toulopoulou T. Working memory circuit as a function of increasing age in healthy adolescence: a systematic review and meta-analyses. Neuroimage Clin. 2016;12:940–8. 10.1016/j.nicl.2015.12.002. 27995059 10.1016/j.nicl.2015.12.002 PMC5153561

[35] Owen AM, mcmillan KM, Laird AR, Bullmore E. N-back working memory paradigm: a meta-analysis of normative functional neuroimaging studies. Hum Brain Mapp. 2005;25:46–59. 10.1002/hbm.20131. 15846822 10.1002/hbm.20131 PMC6871745

[36] Liu J, Zhang M, Jou J, Wu X, Li W, Qiu J. Neural bases of falsification in conditional proposition testing: evidence from an fmri study. Int J Psychophysiol Off J Int Organ Psychophysiol. 2012;85:249–56. 10.1016/j.ijpsycho.2012.02.011. 10.1016/j.ijpsycho.2012.02.011 22387620

[37] Reverberi C, Cherubini P, Frackowiak RSJ, Caltagirone C, Paulesu E, Macaluso E. Conditional and syllogistic deductive tasks dissociate functionally during premise integration. Hum Brain Mapp. 2010;31:1430–45. 10.1002/hbm.20947. 20112243 10.1002/hbm.20947 PMC6871069

[38] Cavanna AE, Trimble MR. The precuneus: a review of its functional anatomy and behavioural correlates. Brain J Neurol. 2006;129:564–83. 10.1093/brain/awl004. 10.1093/brain/awl004 16399806

[39] Ravizza SM, Delgado MR, Chein JM, Becker JT, Fiez JA. Functional dissociations within the inferior parietal cortex in verbal working memory. NeuroImage. 2004;22:562–73. 10.1016/j.neuroimage.2004.01.039. 15193584 10.1016/j.neuroimage.2004.01.039

[40] Vaqué-Alcázar L, Sala-Llonch R, Abellaneda-Pérez K, Coll-Padrós N, Valls-Pedret C, Bargalló N, et al. Functional and structural correlates of working memory performance and stability in healthy older adults. Brain Struct Funct. 2020;225:375–86. 10.1007/s00429-019-02009-1. 31873799 10.1007/s00429-019-02009-1

[41] Shah P, Plitman E, Iwata Y, Kim J, Nakajima S, Chan N, et al. Glutamatergic neurometabolites and cortical thickness in treatment-resistant schizophrenia: implications for glutamate-mediated excitotoxicity. J Psychiatr Res. 2020;124:151–8. 10.1016/j.jpsychires.2020.02.032. 32169688 10.1016/j.jpsychires.2020.02.032

[42] Anderson VM, Goldstein ME, Kydd RR, Russell BR. Extensive gray matter volume reduction in treatment-resistant schizophrenia. Int J Neuropsychopharmacol. 2015;18:pyv016. 10.1093/ijnp/pyv016. 25716781 10.1093/ijnp/pyv016 PMC4540109

[43] Cilia B-J, Eratne D, Wannan C, Malpas C, Janelidze S, Hansson O, et al. Associations between structural brain changes and blood neurofilament light chain protein in treatment-resistant schizophrenia. Aust N Z J Psychiatry. 2025;59:248–59. 10.1177/00048674241307906. 39754499 10.1177/00048674241307906

